# Neurovaskuläre Interposition-Femur-Periost-Lappenplastik bei akzidenteller Bolzenschussverletzung der Hand

**DOI:** 10.1007/s00113-022-01209-5

**Published:** 2022-07-04

**Authors:** Andrej Ring, George Augustin Udrescu, Sebastian Ulrich Bushart, Niklas-Chris Dellmann, Timur Sadykov, Mathias Witt

**Affiliations:** Klinik für Plastische Chirurgie, SLG St. Paulus GmbH, St. Rochus Hospital, Glückaufstr. 10, 44575 Castrop-Rauxel, Deutschland

## Anamnese

Ein 53-jähriger Patient zog sich beim Reinigen eines Bolzenschussgerätes zur unterirdischen Kleintierjagd eine Verletzung an der linken Hand zu, als sich plötzlich ein Schuss löste. Begleitverletzungen lagen nicht vor. Nebenerkrankungen bestanden nicht. Eine Auffrischung des Tetanusschutzes wurde bei Ankunft in der Notfallaufnahme umgehend durchgeführt.

## Befund und Diagnose

**Figure 1:**
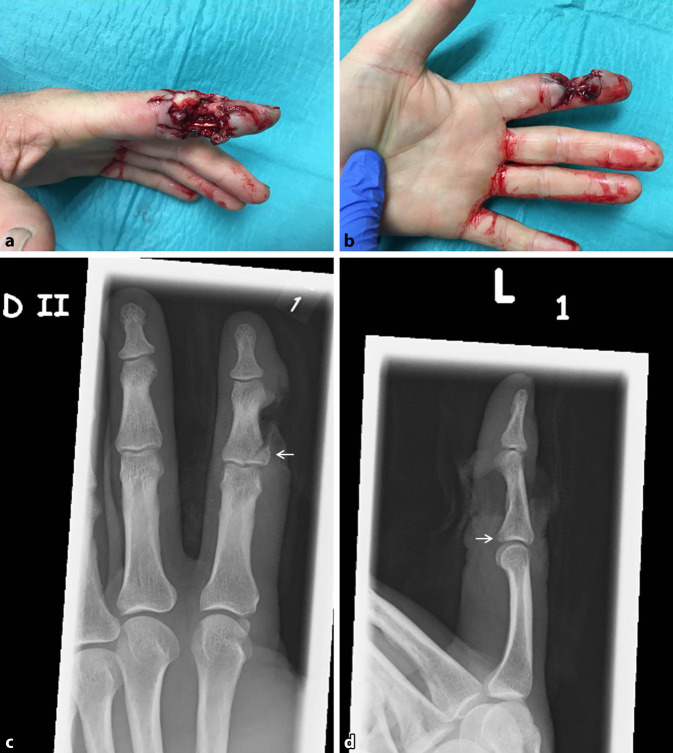

Es fand sich am Zeigefinger eine 3 × 4 cm messende Risswunde mit einem ausgedehnten Haut-Weichteil-Defekt radial über dem Mittelgelenk und -glied (Abb. 1a,b). Die Durchblutung des Endgliedes am Zeigefinger war erhalten, bei radial fehlender Sensibilität distal der Verletzung und erhaltener Flexion und Extension der benachbarten Gelenke.

Nativradiologisch bestand eine schräg verlaufende Basisfraktur des Mittelgliedes des Zeigefingers, radialseitig, mit Abgrenzung eines keilförmigen Ausrissfragmentes ohne relevante Stufenbildung der Gelenkfläche (Abb. 1c,d).

Die potenziell mit Erdkeimen und Tierresten verschmutzte Schusswunde wurde im Rahmen des notfallmäßigen explorativen Débridements gereinigt. Es lag eine langstreckige Zerstörung des 3. Gefäß-Nerven-Bündels mit Substanzverlust der neurovaskulären Strukturen vor.

Nach erfolgter Nekrosektomie am Unfalltag wurden eine Fadenmarkierung der Gefäß-Nerven-Stümpfe durchgeführt, die 3° offene Mittelgliedbasisfraktur durch K‑Draht-Osteosynthese versorgt und der Haut-Weichteil-Defekt bei freiliegenden Knochen und Beugesehnen mit einem synthetischen Hautersatz temporär abgedeckt.

## Therapie und Verlauf

Im Anschluss an die Erstversorgung wurde der intraoperative Befund mit dem Verletzten ausführlich besprochen und eine plastische Rekonstruktion unter Berücksichtigung der expliziten Wünsche des Patienten hinsichtlich einer schnellen Genesung und beruflichen Wiedereingliederung mit möglichst maximalem Funktionserhalt der Hand für die weitere Berufsausübung als selbstständiger Garten- und Landschaftsbauer für den Folgetag (am 3. Tag nach Unfall) geplant.

**Figure 2:**
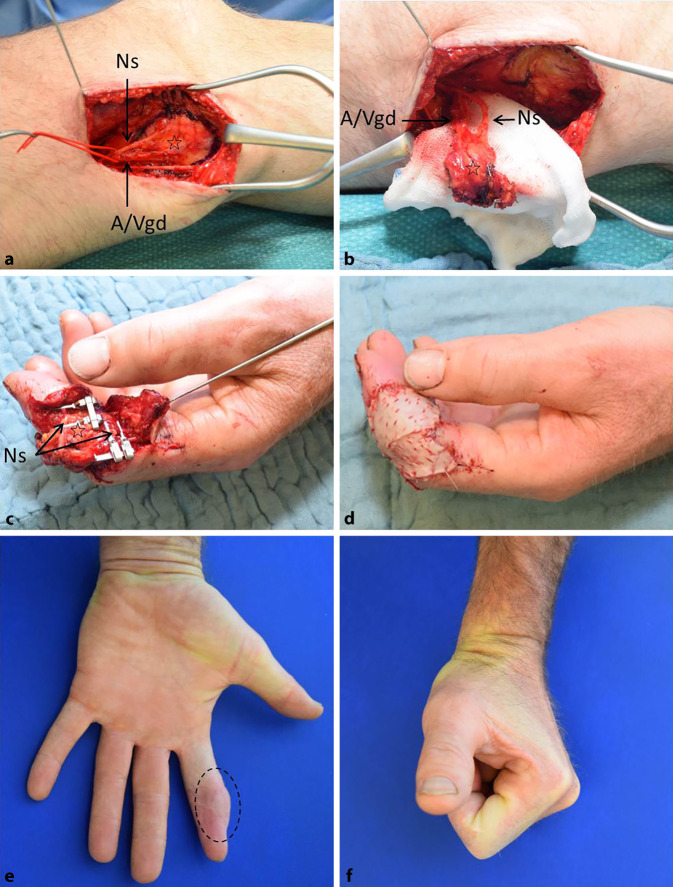

Für die einzeitige Rekonstruktion des Defektes am Zeigefingers wurde ein freier Femur-Periost-Lappen vom ipsilateralen Oberschenkel verwendet (Abb. 2a,b). Die mikrovaskuläre Anastomosierung des von der A. und V. genicularis descendens (A/Vgd) versorgten Transplantats erfolgte arteriell End-zu-End an den zurückgekürzten proximalen Stumpf der verletzten A. digitalis (A3). Für die venöse Anastomose wurde eine dorsale Subkutanvene verwendet. Die Rekonstruktion des verletzten N. digitalis (N3) erfolgte mit dem in den Lappen integrierten Nerveninterponat eines ventralen Astes des N. saphenus (Abb. 2c). Der Femur-Periost-Lappen wurde anschließend mit einem Vollhauttransplantat, welches vom Wundrand des Hebedefektes am Oberschenkel entnommen wurde, abgedeckt (Abb. 2d).

Die Wundheilung verlief unkompliziert. Am 6. Tag nach der Rekonstruktion wurde der Patient in die weitere ambulante Behandlung entlassen. Nach Ablauf von 5. Wochen wurde eine K‑Draht-Entfernung bei vollständiger knöcherner Konsolidierung vorgenommen. Die ambulante Nachbeobachtung erfolgte danach einmal monatlich. Funktionelle Einschränkungen wurden im weiteren Verlauf nicht beklagt. Es bestand eine Schutzsensibilität über der radialen Seite der Fingerkuppe 4 Monate nach der Rekonstruktion (Abb. 2e,f).

## Diskussion

Die Rekonstruktion substanzieller Defekte stellt unter Berücksichtigung des funktionellen Endergebnisses eine Herausforderung für die Handchirurgie dar. Die Operationstechnik des hier beschriebenen neurovaskulären Interposition-Femur-Periost-Lappens entspricht bis auf wenige Modifikationen der Technik, die von Saad et al. vorgestellt wurde [3]. Diese entwickelte sich wiederum als Modifikation des freien medialen Femurkondylentransplantats von Bakri et al. [1], der die Anwendung des vaskularisierten Knochentransplantats aus der medialen Femurkondyle bei der Behandlung von Pseudarthrosen und Osteonekrosen an oberen und unteren Extremitäten erstmalig beschrieb [1]. Das Periost der medialen Femurkondyle und der suprakondylären Region wird von Ästen der absteigenden Genikulararterie versorgt. Die Operationstechnik und die ersten Resultate wurden zum ersten Mal im Rahmen des Mayo-Clinic-and-Chang-Gung-Symposiums für Rekonstruktive Chirurgie im Oktober 2016 in München vorgestellt [4]. Die überzeugenden Ergebnisse nachfolgender Rekonstruktionen an der Hand ermöglichten die Ausweitung der Indikation auch auf Defekte an der unteren Extremität [2].

Die hervorragende Perfusion des Femur-Periost-Lappens trug im vorgestellten Fall zur zeitgerechten Frakturkonsolidierung bei und ermöglichte eine optimale Wiederherstellung des Gleitlagers über dem Bandapparat am Gelenk und den Beugesehnen. Weitere Korrektureingriffe wie z. B. zur Kontrakturauflösung oder zur Lappenausdünnung waren nicht notwendig. Die Morbidität der Hebestelle erwies sich als sehr gering. Mit physiotherapeutischer Beübung konnte zeitgerecht suffizient begonnen werden. Eine funktionell maximale Wiederherstellung der Handfunktion konnte erreicht werden.

## Fazit für die Praxis

Der neurovaskuläre Interposition-Femur-Periost-Lappen erlaubt eine suffiziente und stabile Rekonstruktion sowohl des Weichteildefektes als auch des Sehnengleitlagers, sogar bei neurovaskulärem Substanzverlust. Der Lappen bietet den Vorteil einer dünnen, geschmeidigen Weichteilbedeckung und ermöglicht eine unmittelbare postoperative Mobilisation und Rehabilitation, wodurch das funktionelle Outcome maximiert werden kann.

## References

[1] Bakri K, Shin AY, Moran SL (2008). The vascularized medial femoral corticoperiosteal flap for reconstruction of bony defects within the upper and lower extremities. Semin Plast Surg.

[2] Ring A, Beutel H, Udrescu GA, Farzaliyev F (2021). Mikrovaskuläre Rekonstruktion von Defekten am Fuß durch freien Femur-Periostlappen. Orthopade.

[3] Saad NH, Pontell ME, Winters BS, Daniel J, Saad A (2017). The periosteal medial femoral condyle free flap: a new option for soft tissue reconstruction of the distal lower extremity. Ann Plast Surg.

[4] Udrescu G, Ring A (2016). Functional reconstruction of complex defects by vascularized femoral periosteum flap in hand surgery.

